# Ligand Binding Introduces Significant Allosteric Shifts in the Locations of Protein Fluctuations

**DOI:** 10.3389/fmolb.2021.733148

**Published:** 2021-09-01

**Authors:** Ambuj Kumar, Robert L. Jernigan

**Affiliations:** Roy J. Carver Department of Biochemistry, Biophysics and Molecular Biology, Iowa State University, Ames, IA, United States

**Keywords:** elastic network model, Gaussian network model, structural fluctuations, GroEL, allostery, ligand binding, thermodynamics of protein binding

## Abstract

Allostery is usually considered to be a mechanism for transmission of signals associated with physical or dynamic changes in some part of a protein. Here, we investigate the changes in fluctuations across the protein upon ligand binding based on the fluctuations computed with elastic network models. These results suggest that binding reduces the fluctuations at the binding site but increases fluctuations at remote sites, but not to fully compensating extents. If there were complete conservation of entropy, then only the enthalpies of binding would matter and not the entropies; however this does not appear to be the case. Experimental evidence also suggests that energies and entropies of binding can compensate but that the extent of compensation varies widely from case to case. Our results do however always show transmission of an allosteric signal to distant locations where the fluctuations are increased. These fluctuations could be used to compute entropies to improve evaluations of the thermodynamics of binding. We also show the allosteric relationship between peptide binding in the GroEL trans-ring that leads directly to the release of GroES from the GroEL-GroES cis-ring. This finding provides an example of how calculating these changes to protein dynamics induced by the binding of an allosteric ligand can regulate protein function and mechanism.

## Introduction

Many proteins have intrinsic dynamics that relates to their function, with a specific dynamics that enables them to undergo large conformational transitions in response to external stimuli, such as ligand binding (Skjærven et al., 2011). This binding can cause changes to the protein structure, and because the proteins are densely packed, these can show allosteric changes at sites distant from the binding site. Such allosteric communication within a protein is essential for the progression of biochemical processes. Understanding protein allosteric behavior induced by ligand binding is important for understanding the thermodynamics of binding, since these distant flexibilities are representative of an entropy that has rarely been considered in treating protein binding. This is likely important for protein assembly into machine-like structures as well as for investigations of drug binding to target proteins. It has been shown that the binding of small molecules to a protein is associated with increase in protein thermostability (Fukada et al., 1983; Brandts and Lin, 1990; Shrake and Ross, 1990, 1992) and allosterically associated with large conformational transitions in protein (Pan et al., 2000). A systematic study of such conformational transitions induced by ligand binding can also yield mechanistic insights, as will be shown here for the chaperonin GroEL.

It is well accepted that binding a ligand to a protein causes a gain in enthalpy because of the new interactions formed, which can then be cancelled, at least in part, by a loss in entropy at the binding site, leading to a smaller net change in the free energy of the system. Binding of a ligand is normally favored by the change in residue fluctuations at the protein binding site. This is a type of enthalpy-entropy compensation (EEC), and it has been used to understand the thermodynamic changes involved in ligand binding (Gallicchio et al., 1998; Talhout et al., 2003; Lafont et al., 2007; DeLorbe et al., 2009; Ryde, 2014). It has also been suggested that a stronger interaction between a ligand and a protein will cause a relatively larger loss in entropy in comparison with resulting weaker ligand-protein interactions (Gallicchio et al., 1998). Detailed studies on EEC in ligand recognition and binding has been extensively discussed and reviewed (Dunitz, 1995; Brownowska, 2011; Reynolds and Holloway, 2011; Chodera and Mobley, 2013). EEC calculations for binding of a large number of related ligand molecules show clear evidence of EEC, where no change in the binding affinity was observed despite the large change in ΔH as well as–TΔS of the system, where ΔH is the change in enthalphy, T is the temperature and ΔS is the change in entropy (Talhout et al., 2003). Another study showed a linear relationship between ΔH and TΔS with evidence of EEC upon binding of Ca^2+^ ion to calmodulin (Kuroki et al., 1992), which has been linked to a folding-like process. Such evidence suggests that EEC changes upon ligand binding can influence protein folding and reorganization of residue contacts.

The conformational changes upon ligand binding are accompanied by changes in the protein packing, which affects its stability, as manifested in changes to the fluctuation spectrum. These changes in fluctuations in a protein structure are changes correlated with the binding process itself. It has been proposed that the residual motions of the protein-ligand system after binding can be estimated with vibrational entropy (Dunitz, 1995). Such vibrational motions can enable the transfer of motion across the distant parts of a protein. Therefore, it is evident that local EEC changes induced by ligand binding can cause other changes to be transmitted across the protein, leading to the losses as well as gains in fluctuations, even away from the binding site. The Cooper-Dryden model (Cooper and Dryden, 1984) considers the importance of entropy in the allosteric regulation of protein mechanism, proposing a role for the shift in vibrational motions in mediating allosteric responses. It has been shown that the changes due to ligand binding can be transmitted to the distant parts of the protein through changes in atomic fluctuations (Cooper and Dryden, 1984). The idea of entropy compensation has been further validated where the change in fluctuation at the ligand binding site has been shown to induce increased fluctuations in physically distant parts of a protein (Müller et al., 1996). Moreover, binding of a ligand leads to some redistribution of conformational entropy, which plays an important role in regulating protein mechanism (Capdevila et al., 2017). Nevertheless, the role of such allosteric changes associated with fluctuations and conformational entropy remain poorly understood. Below, we demonstrate how entropy can almost be viewed to be somewhat conserved before and after ligand binding and that this can have a significant impact on assessment of the thermodynamics of the binding process. Our results demonstrate that ligand-induced increases in fluctuations in distant parts of the protein are important and may be critical for a better understanding of protein binding.

## Method

### Data Collection

Ligand bound structures were downloaded from the PDB database (Berman et al., 2002). The unbound structures are generated by removing the ligands from these structures since there are few unbound structures. The GroEL-GroES-peptide bound structure was generated by placing peptides into the GroEL-GroES-ADP structure (PDB ID: 1PF9) at their locations in the peptide bound GroEL structure (PDB ID: 1MNF), to minimize the local RMSD fit. Visualization uses Pymol (Vinet and Zhedanov, 2011).

### Gaussian Network Model

Elastic Network Models are a simple way to obtain the important protein dynamics. While they may not provide all of the details of atomic molecular dynamics, it has been demonstrated as a reliable way to characterize the overall dynamics of proteins, particularly for the slowest motions that are the most important for function, which are difficult to obtain with molecular dynamics. The earliest such model is still the best type in agreeing best with crystallographic B factors and is used here to characterize the locations of the part of the structure showing the largest fluctuations and is clearly sufficient to demonstrate the large shifts in the location of the most conformationally labile part of the structure. This Elastic Network Model used here is the Gaussian network model (GNM) (Bahar et al., 1997; Rader et al., 2005) that was developed to study the collective motions in terms of the scalar fluctuations for individual amino acids. This model is distinguishable from the commonly used ANM that provides information about the directions of motion that are essential for understanding dynamics. Here we implement this GNM in the customary way on coarse-grained proteins by using only the C^α^ atoms to represent each amino acid. All C^α^ atoms and ligand atoms within a distance of 7.5 Å are connected with identical Hookean springs having the same spring constant. The energy for GNM is given as

$$\begin{array}{c} V=\frac{1}{2}\gamma \sum_{i,j}^{N}\text{Γ}\left[{\left(\Delta R_{i}-\Delta R_{j}\right)}^{2}\right] \end{array}$$ (1)

where $\Delta R_{i}$ and $\Delta R_{j}$ are the fluctuation vector of amino acids i and j, γ is the spring constant, Γ is an N×N symmetric matrix, which specifies the node connectivity, where the springs are placed in the protein, and is defined as

$$\begin{array}{c} \Gamma =\{\begin{array}{c} -1\;\;\;\;\;\;\;\;\;\;if\;i\neq j\;and\;R_{ij}\leq r_{c}\\ 0\;\;\;\;\;\;\;\;\;\;\;\;if\;i\neq j\;and\;R_{ij}>r_{c}\\ -\underset{i,\;i\;\neq j}{\sum }{\text{Γ}}_{ij}\;\;if\;i=j\; \end{array} \end{array}$$ (2)

Singular value decomposition yields the eigenvector (υ) and eigenvalues (λ) of Γ. Fluctuations of each residue i ($f_{i}$) are calculated as

$$\begin{array}{c} f_{i}=\sum_{m}\frac{\upsilon_{mi}^{2}}{\lambda_{m}} \end{array}$$ (3)

where, $\upsilon_{mi}$ is the eigenvector of residue i mode m and $\lambda_{m}$ is the eigenvalue of mode m. Differences in residue fluctuations due to ligand binding are calculated from

$$\begin{array}{c} \Delta f_{i}=\;f_{i}^{\left(perturbed\right)}-f_{i}^{\left(normal\right)} \end{array}$$ (4)

where, $f_{i}^{\left(perturbed\right)}$ is the fluctuation of residue i when ligand is bound to the protein and $f_{i}^{\left(normal\right)}$ is the fluctuation of residue i for the protein with no ligand bound. Note that $\Delta f_{i}$ can have either positive or negative values, indicating either an increase in fluctuations in residue i or its reduced mobility. As will be seen, the localization of fluctuations is drastically impacted by the binding of ligand. While it is readily apparent that fluctuations at the location of the bound ligand are reduced by its specific interactions, it has not been so widely accepted that ligand binding induces specific remote increases in fluctuations. As we show below in several examples, the locations for these changes are readily discerned with the simple Gaussian Network Model. As we will see, the repositioned fluctuations have positions that depend strongly on the structures themselves.

## Results and Discussion

The resulting changes in the fluctuations (and the corresponding entropies) accompanying binding can be quite different and depend on the details of the protein structure. This work also points up the importance of estimating entropies of proteins, which has always been difficult. The results here suggest that the computed fluctuations could be used directly to estimate the entropies.

**FIGURE 1:**
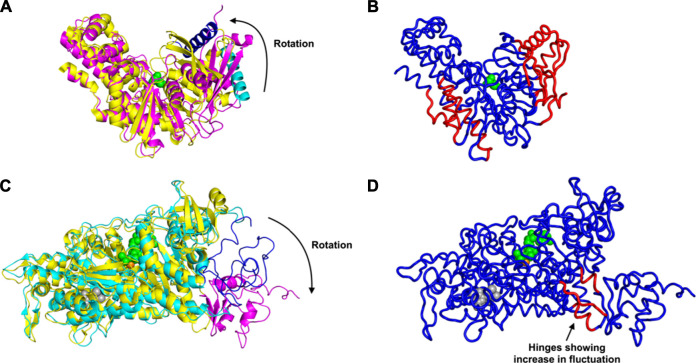
Conformational Transition and Changes in Fluctuations Induced by Ligand Binding, for glucokinase shown in **(A)** as superimposed unbound and bound structures and **(B)** bound. And similarly for the myosin motor protein in **(C)** unbound and bound structures superimposed and **(D)** bound. **(A)** Rotation and domain reorganization upon binding and release of glucose (shown as green spheres). Glucose bound closed form (PDB ID: 1V4S) is shown in magenta and the open form (PDB ID: 1V4T) after glucose is released in yellow. Glucokinase small domain helix residues 117–137 are shown in blue in closed form and in cyan in open form to highlight the domain rotation.**(B)** Increased fluctuations (red) and decreased fluctuations (blue) upon binding of glucose to glucokinase. **(C)** Rotation of the C-terminus (residues 692–776) of the myosin motor protein upon ligand binding. C-terminus residues 692–776 are shown in magenta in the ligand bound form (2JHR) and in blue in the apo structure (PDB: 2Y0R). The ligand ADP metavanadate is shown in green spheres, magnesium ion as an orange sphere, and pentabromopseudilin as grey spheres. **(D)** Sites having increased (red) and decreased (blue) fluctuations in the myosin motor protein upon ligand binding. In the case of the central binding of glucose to glucokinase, these increased fluctuations are on the two outside parts of the structure but in the myosin motor protein, they are instead at the central hinge, indicating a kind of dynamic flexibility between the two domains of this protein.

First we consider the case of glucokinase that regulates the metabolism of glucose; it undergoes a large conformational transition and reorganization within its small domain upon binding of glucose (Kamata et al., 2004) (Figure 1A). The glucose binding induced conformational transition mediates the catalytic cycle of glucokinase (Kamata et al., 2004). This involves multiple steps of conformational changes as well as an intra-domain sliding motion that breaks and forms new contacts within the domain (Kamata et al., 2004). Our results indicate a significant increase in the fluctuations within the glucokinase small domain (on the right side of the structures shown in Figure 1B), which undergoes significant atomic displacement upon ligand binding (Kamata et al., 2004), with a relatively large conformational change as can be seen in Figure 1B, in comparison with the structure of that domain from Figure 1A. Increased fluctuations within this small domain likely assist in the repositioning of these residues and the relatively large conformational changes observed.

Even more striking are the changes observed in the myosin motor protein shown in the unbound form in Figure 1C and in the bound form in Figure 1D. In this case what had been a relatively extended and loosely pack small domain on the right side has become more densely packed and more structured, but the increases in fluctuations after binding are seen in the hinge region between the large and small domains (in red in Figure 1D).

An alternate mechanism of conformational changes induced by ligand binding can be associated with changes in fluctuations within inter-domain hinges. The myosin motor protein has 2,116 residues with the myosin motor at its N-terminal region. It contains a reactive thiol region in the C-terminus of the motor domain comprised of the SH1 (residues 681–689) and SH2 (residues 669–678) helices (Preller et al., 2011). The binding of an allosteric ligand to the motor protein introduces rotation in the C-terminal residues 692–776 (Figure 1C). Our results indicate that binding of an allosteric ligand and ATP together increases the fluctuations in the hinge region at residues 490–496, 499–506, and 686–691, which also contains a part of the SH2 domain (Figure 1D). Increased fluctuations in the hinge region may initiate the rotation of myosin motor domain C-terminal residues following ligand binding.

**FIGURE 2:**
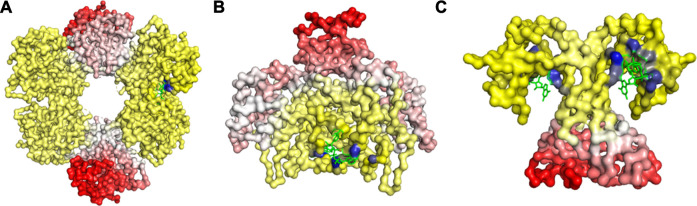
Changes in Fuctuations upon Ligand Binding in **(A)** UDP-glucose 6-dehydrogenase (PDB ID: 5TJH) bound with uridine-5’-diphosphate-glucose, **(B)** Citrate synthase (PDB ID: 4JAF) bound with 1, 4-Dihydronicotinamide adenine dinucleotide, and **(C)** uncharacterized protein VCA0042 (PDB ID: 2RDE) bound with 9,9’-[(2R,3R,3aS,5S,7aR,9R,10R,10aS,12S,14aR)-3,5,10,12-tetrahydroxy-5,12-dioxidooctahydro-2H,7H-difuro[3,2-d:3’,2’-j][1,3,7,9,2,8]tetraoxadiphosphacyclododecine-2,9-diyl]bis(2-amino-1,9-dihydro-6H-purin-6-one). The gain in fluctuations is scaled between 0 (shown in white) and 1 (red), and the loss in fluctuations is scaled between 0 (yellow) and −1 (blue). Here, the ligand is in green.

A trend of decay in change of fluctuation was observed as we move farther from the ligand binding pocket (Figure 2), which was then followed by an increase in fluctuation beyond a certain distance from the binding pocket (Figure 2). These changes indicate that the EEC follows a progressive transmission of fluctuation changes, where the ligand binding pocket experiences a large loss in fluctuations, first leading to relatively lower fluctuation losses in the residues close to the binding site, which is then followed by slight increases in fluctuations as we move farther from the binding site. This trend in fluctuation changes may help us locate the group of residues that are susceptible to conformational transitions upon ligand binding.

**FIGURE 3:**
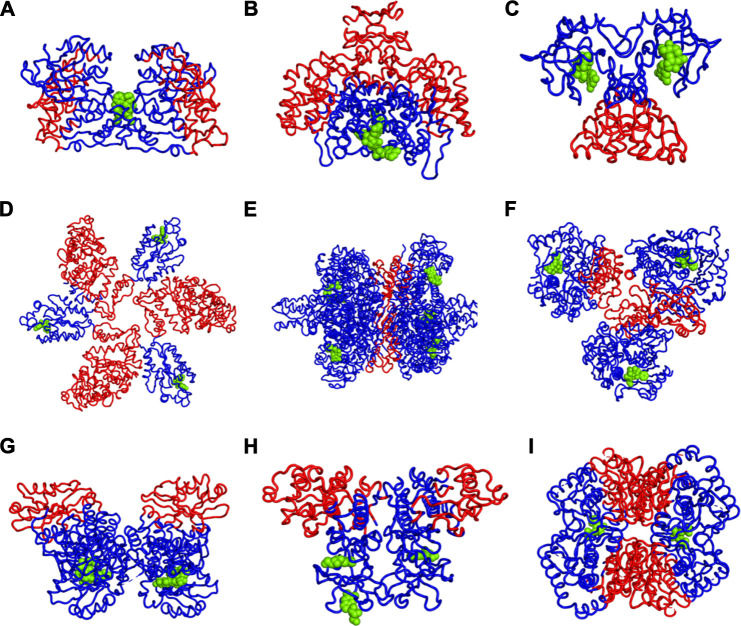
Increases (red) and Decreases (blue) in Fuctuations upon Lgand (green spheres) Binding in **(A)** Mitogen-activated protein kinase 8, **(B)** Citrate synthase, **(C)** Uncharacterized protein VCA0042, **(D)** ATP sulfurylase, **(E)** Glutamate dehydrogenase, **(F)** Acetyl-Coenzyme A carboxylase, **(G)** Isocitrate dehydrogenase kinase/phosphatase, **(H)** Casein kinase II subunit alpha, **(I)** l-lactate dehydrogenase. While there appears to be a large variety of positions that are affected, the universal rule appears to be that the part of the structure furthest away from the binding site has the largest increases in fluctuations. If the binding site is near the outside of the protein, then the largest increases in fluctuations will be at the center of the protein. One particularly interesting such case is shown in part d where there are three occupied binding sites, which can be presumed, each to increase the fluctuations in the most distant part so with the three occupied binding sites all three domains have increased fluctuations near the center of the overall structure.

We have further investigated the allosteric impact of ligand binding for a variety of proteins (Table 1) showing less detail in the values of the fluctuations, such as Mitogen-activated protein kinase 8 (Figure 3A), Citrate synthase (Figure 3B), Uncharacterized protein VCA0042 (Figure 3C), ATP sulfurylase (Figure 3D), Glutamate dehydrogenase (Figure 3E), Acetyl-Coenzyme A carboxylase (Figure 3F), Isocitrate dehydrogenase kinase/phosphatase (Figure 3G), Casein kinase II subunit alpha (Figure 3H), and l-lactate dehydrogenase (Figure 3I) and colored in a simple binary way to indicate only the changes in blue showing all residues with losses fluctuations and red for all residues showing gains in fluctuation. Results consistently indicate increased fluctuations in the distant regions of each protein far from the location where the ligand is bound. In ATP Sulfurylase, binding of the ligand to the C-terminal region shows increases in the fluctuations in the N-terminal region for all three chains (A, B, and C), indicating a compensatory mechanism of entropy conservation across the protein assembly through changes in fluctuations induced by the ligand binding. A similar trend of fluctuation changes in the distant region of the proteins was observed for all the nine structures (Figure 3).

**TABLE 1:** Proteins used in this study together with their corresponding PDB IDs and their ligands used to calculate residue fluctuations in the bound state. Here ligand 46A represents N-butyl-4,6-dimethyl-N-{[2’-(2H-tetrazol-5-yl)biphenyl-4-yl]methyl}pyrimidin-2-amine, NAI represents 1, 4-Dihydronicotinamide adenine dinucleotide, C2E represents 9,9’-[(2R,3R,3aS,5S,7aR,9R,10R,10aS,12S,14aR)-3,5,10,12-tetrahydroxy-5,12-dioxidooctahydro-2H,7H-difuro[3,2-d:3′,2′-j][1,3,7,9,2,8]tetraoxadiphosphacyclododecine-2,9-diyl]bis(2-amino-1,9-dihydro-6H-purin-6-one), PPS represents 3’-Phosphate-adenosine-5’-phosphate sulphate, GTP represents Guanosine-5’-triphosphate, S1A represents Soraphen A, ATP represents Adenosine-5’-triphosphate, AMP represents Adenosine monophosphate, MG represents magnesium ion, RFZ represents 5,6-dichloro-1-beta-d-ribofuranosyl-1H-benzimidazole, and FBP represents 1,6-di-O-phosphono-beta-d-fructofuranose.

Protein	PDB ID	Ligand used
Mitogen-activated protein kinase 8	3O2M	46A
Citrate synthase	4JAF	NAI
Uncharacterized protein VCA0042	2RDE	C2E
ATP sulfurylase	1M8P	PPS
Glutamate dehydrogenase	3ETE	GTP
Acetyl-Coenzyme A carboxylase	1W96	S1A
Isocitrate dehydrogenase kinase/phosphatase	3EPS	ATP, AMP, MG
Casein kinase II subunit alpha	3H30	RFZ
l-lactate dehydrogenase	2LDB	FBP

To address the issue of possible artifacts, we have replaced all protein bonded ligand atoms with a decoy ligand modelled by picking one random atom from all the bonded ligands. The ncreases in fluctuation of the protein residues for the bound known ligand and this artificial decoy ligand binding have been compared to check whether a similar trend of increased fluctuations are also observed for the decoy ligand. The result indicates that decoy ligand induced only a significantly smaller increase in fluctuations in the same regions where increases in fluctuations were observed in response to the known allosteric ligand binding (Table 2). Moreover, in a few cases such as isocitrate dehydrogenase kinase/phosphatase and casein kinase II subunit alpha, binding of this decoy ligand caused larger reductions in the fluctuation gains at the distant region of interest (Table 2). This trend of relatively lower impact of the decoy ligand indicates that the enthalpy-entropic changes observed in our calculations are specific to the actual ligand and are not a general result for any possible ligand. Furthermore, the ratio of mean of increase in fluctuation to the mean of decrease in fluctuation across all residues in the protein upon the actual known ligand binding shows a diverse trend across 12 proteins (Table 2), ranging from a very low value of 0.59% to a very high value of 98.57%, indicating that enthalpy-entropy compensation can occur across a broad range in different proteins, which all suggests that the extent energy-entropy compensation depends on the details of each case, and that it would almost never be exact compensation. Nonetheless, usually the net result of loss of fluctuations (entropy loss) is normally partially compensated by fluctuations (and entropy gains) at distant regions of the protein.

**TABLE 2:** Ratios of increases in fluctuations to decreased in fluctuations upon ligand binding, mean increases in fluctuations in the residues where fluctuation increases upon ligand binding, and mean decrease in fluctuation in the residues where fluctuation decreases upon ligand binding.

PDB ID	Ratio of fluctuation change (%)	Mean increases in flucutations	
		Known ligand	Artificial decoy
4JAF	8.27	0.0014	4.43 × 10^−7^
3O2M	10.60	0.0023	3.01 × 10^−5^
5TJH	98.57	0.0219	0.0009
1V4S	20.61	0.0033	0.0003
2RDE	52.79	0.0326	5.93 × 10^−5^
1M8P	10.19	0.0004	6.50 × 10^−5^
3ETE	0.59	4.23 × 10^−5^	9.78 × 10^−7^
1W96	7.48	0.0009	1.61 × 10^−5^
3EPS	6.63	0.0023	−7.12 × 10^−6^
3H30	9.13	0.0022	−6.64 × 10^−6^
2LDB	52.20	0.0075	0.0001
2JHR	3.6	0.0005	4.66 × 10^−6^

**FIGURE 4:**
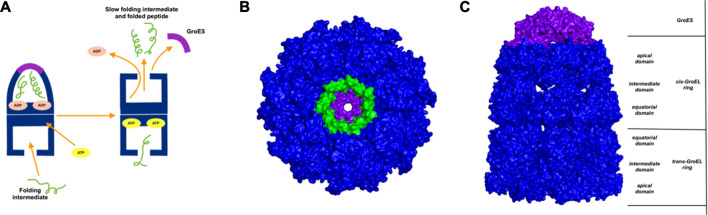
Release of peptide ligand from GroEL-GroES Complex. **(A)** Mechanism of GroEL-GroES cis-ring uncapping is likely mediated by trans-ring peptide binding. GroEL is shown in blue and GroES in purple and peptide in green. **(B)** The bottom GroES trans-ring binds with peptide. **(C)** Perpendicular view of GroEL-Groel assemblage.

GroEl is a chaperone protein essential for the proper folding of various misfolded proteins. GroEL contains two rings composed of seven subunit each, connected back-to-back to form a central substrate binding chamber (Figure 4). One GroEL ring can also bind to the GroES cap, a co-chaperone protein closing over the GroEL, to create a cis-ring complex. The GroEL-GroES cis-ring binds to ATP and undergoes biomechanical motions that pull folded proteins apart to enable them to refold. Many experiments have been conducted to understand the mechanism involved in GroEL-GroES based chaperone folding under conditions where the spontaneous folding mechanism fails to proceed (Weissman et al., 1994; Fenton and Horwich, 1997; Sigler et al., 1998; Ishino et al., 2015).

**FIGURE 5:**
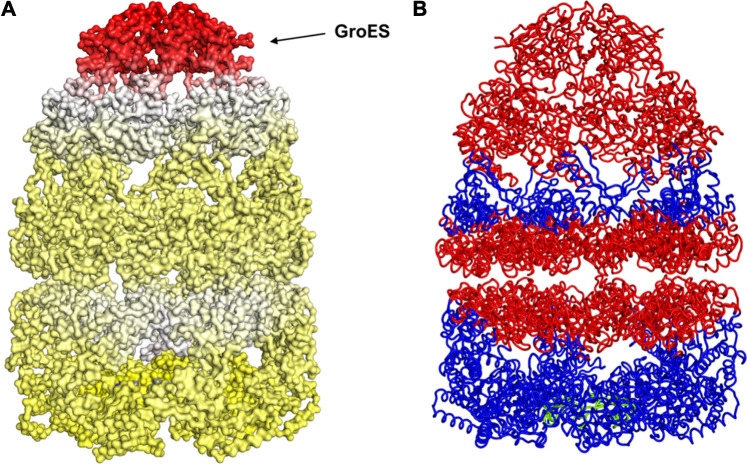
Mechanism for release of GroES (and internal peptide). Change in fluctuations in the Groel-GroES assemblage upon peptide binding. **(A)** Gains of fluctuations are scaled between 0 (white) and 1 (red), and loss of fluctuations shown also but separately between 0 (yellow) and −1 (blue), **(B)** Increased (red) and decreased (blue) fluctuations upon peptide binding is shown. This suggests that peptide binding to the lower ring causes release of GroES and internal peptide from the upper ring.

With the gain of broader insights into the GroEL molecular mechanism, the detailed understanding of its allosteric regulation has seen recent gains. It has been shown that there is direct allosteric coupling between the assembly of the GroEL-GroES cis-ring on one end with the disassembly of the cis-ring on the other end of the protein (Lin and Rye, 2006). Binding of ATP and the peptide to the open trans-ring on a GroEL complex triggers the disassembly of the cis-ring (Lin and Rye, 2006) (Figure 4A). In this work, we are investigating the role of binding of intermediately folded peptide binding to the trans-ring of GroEL to trigger the disassembly of the cis-ring (Figure 4B). Our results show that binding of peptide chains to the trans-ring allosterically increases the fluctuations in the GroEL cis-ring apical domain, in the GroES protein as well as in the equatorial domain of GroEL cis and trans-ring (Figure 5). The increased fluctuations in the GroEL cis-ring apical domain and GroES are likely to affect the binding affinity of GroES cap, facilitating the release of the GroES. The increased fluctuations with the GroES cap itself are likely to play an important role in its unbinding. Moreover, the increase in flexibility in equatorial domain of the cis-ring may also facilitate the release of the bound ADP molecules.

Detailed insights into how ligand binding relates to EEC can help to understand large conformational changes, molecular motions associated with protein function, structure-function relationships, as well as how to better evaluate docked protein poses, which are usually based on enthalpies while ignoring entropies. The extent of energy-entropy compensation appears to be highly variable for different cases as determined by isothermal titration calorimetry (Olsson et al., 2011). It is difficult to predict the effects of small changes; for example, an added hydrogen acceptor group on a HIV-protease inhibitor yielded a gain in enthalpy, but the corresponding loss in entropy compensated and resulted in an insignificant change in the binding affinity of the ligand (Lafont et al., 2007). A similar phenomena was observed where chemical modifications to a thrombin ligand failed to cause significant changes in ligand binding affinity due to competing changes between the enthalpic and entropic contributions (Baum et al., 2010). Such EEC is commonly observed for ligand-protein interactions (Whitesides and Krishnamurthy, 2005). Therefore, a better understanding of the entropic changes induced by ligand binding is needed for more reliable computations of the effects of binding.

## Conclusion

We have demonstrated the allosteric transfer of fluctuations to distant regions of a variety of proteins as differential changes in fluctuations, driven by some level of conservation of entropy throughout the whole protein. These changes in fluctuations may induce a conformational rearrangement within protein domains or cause allosteric conformational transitions. The pattern of ligand binding induced fluctuation changes observed in this study indicates that changes to the local packing density can influence the dynamics of the most distant regions within even the largest protein assemblages such as the example shown here for GroEL and may play a significant role in regulating and controlling their molecular mechanisms.

## Data Availability

The raw data supporting the conclusions of this article will be made available by the authors, without undue reservation.
